# Reduction of dopamine D_2/3_ receptor binding in the striatum after a single administration of esketamine, but not R-ketamine: a PET study in conscious monkeys

**DOI:** 10.1007/s00406-016-0692-7

**Published:** 2016-04-18

**Authors:** Kenji Hashimoto, Takeharu Kakiuchi, Hiroyuki Ohba, Shingo Nishiyama, Hideo Tsukada

**Affiliations:** 1 Division of Clinical Neuroscience, Chiba University Center for Forensic Mental Health, Chiba, 1-8-1 Inohana, Chiba, Chiba 260-8670 Japan; 2 Central Research Laboratory, Hamamatsu Photonics KK, Hamamatsu, Shizuoka 434-0041 Japan

**Keywords:** Dopamine D_2/3_ receptor, Esketamine, Release, R-ketamine, Monkey

## Abstract

R-ketamine appears to be a potent, long-lasting and safer antidepressant, relative to esketamine (S-ketamine), since it might be free of psychotomimetic side effects. Using [^11^C]raclopride and positron emission tomography (PET), we investigated whether esketamine and R-ketamine can affect dopamine D_2/3_ receptor binding in the conscious monkey brain. A single infusion of esketamine (0.5 mg/kg), but not R-ketamine (0.5 mg/kg), caused a reduction of binding availability of dopamine D_2/3_ receptor in the monkey striatum. This study suggests that unlike to R-ketamine, esketamine can cause dopamine release in the striatum, and that its release might be associated with psychotomimetic effects of esketamine.

## Introduction

The rapid-onset antidepressant effects of the *N*-methyl-D-aspartate (NMDA) receptor antagonist ketamine have attracted serious attention after it was found that a single sub-anesthetic dose (0.5 mg/kg) of ketamine elicited a rapid antidepressant effect within 1–2 h, in depressed patients, including those with treatment-resistant depression and treatment-resistant bipolar depression [1–4]. These beneficial effects persist for up to 2 weeks in some patients. A recent meta-analysis demonstrated that ketamine produced a rapid, yet transient, antidepressant effect, with odds ratios for response and transient remission of symptoms at 24 h equaling 9.87 and 14.7, respectively, accompanied by brief psychotomimetic and dissociative effects [5].

Ketamine (or RS-ketamine) is a racemic mixture containing equal parts of R-ketamine and S-ketamine (esketamine). Esketamine shows an approximately threefold to fourfold greater anesthetic potency and greater undesirable psychotomimetic side effects, compared with R-ketamine [6]. This is related to the fact that esketamine has an approximately fourfold greater affinity for the NMDA receptor relative to R-ketamine [6]. We reported that R-ketamine shows greater potency and longer-lasting antidepressant effects than esketamine in animal depression models, including neonatal dexamethasone exposure, repeated social defeat stress and learned helplessness, and that unlike esketamine, R-ketamine does not induce psychotomimetic side effects and abuse potential in rodents [7–9]. In addition, we reported that a single dose of esketamine (10 mg/kg), but not R-ketamine (10 mg/kg), resulted in loss of parvalbumin (PV)-positive cells in mouse brain regions, such as the medial prefrontal cortex [8], suggesting that loss of PV-positive cells may be associated with psychosis.

Dopamine D_2/3_ receptors have a high-affinity state for endogenous dopamine and low-affinity state. Raclopride is a moderately high-affinity selective antagonist at these states of the dopamine D_2/3_ receptors. [^11^C]Raclopride has been used as a positron emission tomography (PET) ligand for characterization dopamine D_2/3_ receptors in the brain from human and monkey. Interestingly, PET using [^11^C]raclopride would be useful for detection of release of endogenous dopamine from presynaptic terminal [10]. Given differential effects of ketamine enantiomers in the brain, the present study using PET was performed to examine whether a single infusion of ketamine enantiomers could affect the release of endogenous dopamine in the conscious monkey brain.

## Methods

### Animals and drugs

Experiments were conducted in accordance with the recommendations of the US National Institutes of Health. The following experiments were approved by the Ethical Committee of the Central Research Laboratory, Hamamatsu Photonics (Hamamatsu, Shizuoka, Japan). Four male rhesus monkeys (Macaca mulatta; 7.1 ± 1.3 years old, weighing 7.2 ± 1.1 kg) were studied 3 times (saline, esketamine and R-ketamine). The order of PET scan was saline, esketamine and R-ketamine with 2-week period between each scan. The doses (0.5 mg/kg) of esketamine or R-ketamine hydrochloride were based on a previous monkey study [11] and human studies [1–4]. Esketamine hydrochloride and R-ketamine hydrochloride were synthesized by K.H. at Chiba University (Chiba, Japan) and were dissolved in physiological saline.

### Preparation of [^11^C]raclopride and PET experiments

[^11^C]Raclopride was labeled by *N*-methylation of respective nor compound with [^11^C]methyl triflate prepared from [^11^C]methyl iodide. The radioactive purity of [^11^C]raclopride was greater than 98 %, and the specific radioactivity was 32.8 ± 5.6 GBq/μmol (mean ± SD, *n* = 12). A high-resolution animal PET scanner (SHR-7700; Hamamatsu Photonics, Hamamatsu, Japan) with a transaxial resolution of 2.6-mm full width half maximum in the enhanced 2D mode and a center-to-center distance of 3.6 mm [12] was used. PET images were reconstructed by a filtered back projection method with a 4.5-mm Hanning filter, resulting in an in-plane reconstructed resolution of 4.5 mm. PET scans with [^11^C]raclopride were performed without arterial blood sampling. The trained animal’s head was rigidly fixed to the upper frame of a monkey chair using an acrylic head-restraining device. The animal sitting in a restraining chair was placed at a fixed position in the PET gantry with stereotactic coordinates aligned parallel to the orbitomeatal line. Transmission data with a ^68^Ge–^68^Ga pin source were obtained for an attenuation correction.

**Fig. 1:**
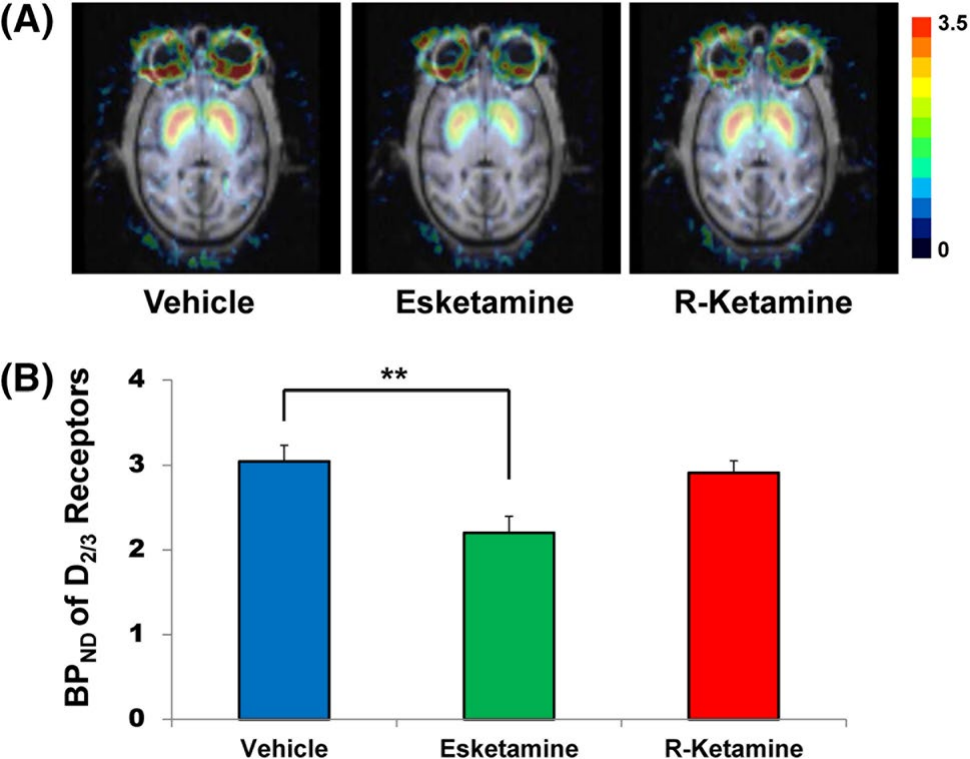
Effect of esketamine and R-ketamine on the BP_ND_ of [^11^C]raclopride to dopamine D_2/3_ receptors in the striatum of conscious monkeys. **a** Representative photomicrographs of typical MRI and parametric PET images of [^11^C]raclopride to dopamine D_2/3_ receptors from vehicle (saline)-treated, esketamine (0.5 mg/kg, 40-min)-treated and R-ketamine (0.5 mg/kg, 40-min)-treated monkeys. PET images from 61 to 91 min were obtained. *Color bar* indicates a level of the non-displaceable binding potential (BP_ND_) of [^11^C]raclopride. **b** The non-displaceable binding potential BP_ND_ of [^11^C]raclopride in the striatum. The data show the mean ± S.E.M. (*n* = 4). **P < 0.01 compared with vehicle (saline)-treated condition

[^11^C]Raclopride was administered intravenously after the end of ketamine infusion (0.5 mg/kg for 40 min), and PET scans were also started after the end of ketamine infusion. Vital signs including heart rate, respiration rate, systolic and diastolic blood pressure, and body temperature were monitored throughout the infusion of ketamine enantiomers. PET scans were acquired for 91 min after intravenous bolus injection of [^11^C]raclopride. The injected dose of [^11^C]raclopride was 164.2 ± 15.9 MBq/kg (mean ± SD, *n* = 12). A summation image from 61 to 91 min post-injection was obtained (Fig. 1). The regions of interest (ROIs) were drown on the individual MRI in bilateral caudates and putamens, and cerebellum, and automatically copied and pasted them on the corresponding PET image slices, then converted these ROIs into one VOI using PMOD. The time-activity curves obtained from the VOI were applied for simplified reference tissue model (SRTM) analysis. The quantitative analysis of [^11^C]raclopride was performed with SRTM in order to calculate the non-displaceable binding potential (BP_ND_) [13] using the time-activity curve in the cerebellum as an input function.

### Statistical analysis

The data are shown as the mean ± standard error of the mean (S.E.M.). Analysis was performed using PASW Statistics 20 (formerly SPSS Statistics; SPSS). Comparisons between groups were made using the repeated-measures analysis of variance (ANOVA), followed by post hoc Bonferroni test. The *P* values of less than 0.05 were considered statistically significant.

## Results

Marked accumulation of radioactivity in the striatum was shown after an intravenous administration of [^11^C]raclopride (Fig. 1). Radioactivity in the striatum of esketamine-treated monkeys was lower than those of vehicle (saline)-treated monkeys (Fig. 1). Repeated-measures ANOVA demonstrated a significant change among three conditions in the striatum (*F* = 70.252, *P* < 0.001). *Post* *hoc* Bonferroni test revealed that a single infusion of esketamine significantly (*P* = 0.004) decreased BP_ND_ in the striatum of monkey brain (Fig. 1). In contrast, a single infusion of R-ketamine did not decrease BP_ND_ in the striatum of monkey brain (*P* = 0.603) (Fig. 1).

## Discussion

Using a conscious PET study, we found that a single infusion of esketamine, but not R-ketamine, elicited a significant reduction of BP_ND_ of dopamine D_2/3_ receptor in the striatum of monkeys. Since [^11^C]raclopride-PET has been used for detection of release of endogenous dopamine from presynaptic terminal [10], this study suggests that esketamine, but not R-ketamine, causes the marked release of endogenous dopamine from presynaptic terminal in the striatum of monkeys.

Clinical use of ketamine is limited due to its side effects such as psychotomimetic effects [14]. Unlike esketamine, R-ketamine might not appear to cause psychotomimetic effects, based on the lack of behavioral abnormalities (e.g., hyperlocomotion, prepulse inhibition deficits) observed in rodents after treatment [8]. Recently, Singh et al. [15] reported a rapid-onset antidepressant effect of esketamine in treatment-resistant patients with depression although Brief Psychiatric Rating Scale (BPRS) score and Clinician Administered Dissociative States Scale (CADSS) score were the highest at 40 min after an infusion of esketamine (0.20 or 0.40 mg/kg for 40 min). Furthermore, an infusion of esketamine, but not R-ketamine, in healthy subjects produced a dissociative state and psychotic syndrome, including disturbances of emotion and sensory perception, difficulties in thinking and reality appraisal, as well as ego disorders [16]. Subsequently, Vollenweider et al. [17] reported that an infusion of esketamine caused the reduction of in vivo binding of [^11^C]raclopride binding to dopamine D_2/3_ receptors in human brain, indicating an increase of dopamine in the striatum. Taken together, it is likely that esketamine-associated psychotomimetic effects in patients might be associated with marked dopamine release in the striatum. Given the role of dopamine release in the striatum for ketamine (or esketamine)-induced psychotomimetic effects, it is unlikely that R-ketamine might cause psychotomimetic effects in monkeys or humans.

Despite an increasing number of studies focusing on the rapid antidepressant effects of ketamine in treatment-resistant depression, its potential to elicit abuse liability cannot be ignored [14]. In contrast, a schedule of repeated ketamine infusions could provide effective management of depressive symptoms in patients with treatment-resistant depression [15]. A study using the conditioned place preference (CPP) test showed that ketamine (1.0–10 mg/kg) significantly increased CPP scores in mice, in a dose-dependent manner [18], suggesting that ketamine has rewarding effects. We also reported that repeated administration of esketamine (5, 10 or 20 mg/kg), but not R-ketamine (5, 10 or 20 mg/kg), significantly increased CPP scores in mice, in a dose-dependent manner [8]. Taken together, it is possible that release of dopamine by esketamine could induce rewarding effects in rodents [8]. Given the role of dopamine release in the striatum for ketamine-induced rewarding effects, it is unlikely that R-ketamine might cause rewarding effects in monkeys or humans. These findings suggest greater safety for repeated intermittent dosing with R-ketamine relative to esketamine.

In conclusion, this study demonstrates that a single infusion of esketamine, but not R-ketamine, could cause a marked reduction of BP_ND_ of dopamine D_2/3_ receptor in the striatum of conscious monkeys. Therefore, relative to esketamine, R-ketamine is likely to be a safer antidepressant in the treatment of depression.
